# Neligan on Diseases of the Skin

**Published:** 1856-09

**Authors:** 


ART. X. — *Neligan on Diseases of the Skin.*

We have received the manual accompanying the Atlas, noticed in a previous number. It is not intended as a full work on the subject, but is a compend which will very much aid the practitioner in this difficult branch of diagnosis.

Taken with the beautiful plates of the Atlas, which are remarkable for their accuracy and beauty of coloring, it constitutes a very valuable addition to the library of the practical man. There is no branch of medical science in which there is so much routinism, and vague indifinite ideas of treatment, as in diseases of the skin. The early writers seem to have taken infinite pains to complicate the subject, by an absurd nomenclature, and we fervently wish that some innovating spirit would topple over the whole fabric of long Greek terminations, and substitute in its place something rational and intelligible.

